# The remote neuro-otology assessment – managing dizziness in the coronavirus disease 2019 era

**DOI:** 10.1017/S0022215120002273

**Published:** 2020-10-21

**Authors:** L Murdin, Y Saman, P Rea

**Affiliations:** 1 ENT Department, Guy's and St Thomas’ NHS Foundation Trust, London, UK; 2 UCL Ear Institute, London, UK; 3 ENT Department, University Hospitals of Leciester NHS Trust, UK

**Keywords:** Dizziness, Vertigo, Telemedicine, Neuro-Otology, Remote Consultations

## Abstract

**Background:**

Coronavirus disease 2019 and other factors have driven interest in conducting remote consultations, but there has been little research on this topic in neuro-otology. With suitable preparation, neuro-otology patients with dizziness can have remote assessments that include elements of neuro-otological physical examination, with tailored management and onward pathways arranged.

**Methods:**

This paper reports experience with remote consultation in over 700 neuro-otology patient consultations and suggests a systematic approach, illustrated by a clinical case report and data on 100 consultations.

**Conclusion:**

Remote consultations can play a role in neuro-otology clinics. Further research is needed to establish patient acceptability, diagnostic accuracy, safety and efficiency of remote models of care for this patient group.

## Introduction

In recent months, telemedicine, as part of a model of care that minimises face-to-face contact between clinician and patient, has been explored in new settings in light of the ongoing coronavirus disease 2019 (Covid-19) pandemic.^1^ The General Medical Council and others have given guidance around remote consultations.^2^ Previous studies show that video consultations are often associated with high satisfaction amongst patients and staff, with no difference in clinical outcomes.^3^

Although tele-audiology with respect to hearing has been trialled,^4,5^ we are not aware of any published reports, trials or research of practising neuro-otology assessments or management through remote consultations. Patients with dizziness and balance problems can find attending hospitals, with the associated need for travel, difficult because of symptom induction. Therefore, remote consulting has the potential to be of specific benefit to this group.

This report describes our approach to remote neuro-otology patient assessment with a case report illustration. It is based on our experience between 2017 and 2020 of over 700 remote consultations. Data from 100 consecutive new patient consultations (50 video and 50 telephone) are summarised in Table 1.

**Table 1.** Data from 100 remote new patient consultations conducted by video and telephone

Parameter	Video (neuro-otology clinic; *n* = 50)	Telephone (ENT balance clinic; *n* = 50)
Sex (females)	64%	72%
Age (years)		
– Mean (SD)	53 (20)	54 (20)
– Range	24–77	21–93
Ethnicity (*n* (%))		
– White	16 (32) (British)	32 (64) (British & other)
– Black	9 (18)	–
– Asian/Indian/other	12 (24)	18 (36) (Asian)
– Missing data	13 (26)	–
Working diagnosis (*n* (%))		
– BPPV	11 (22)	13 (26)
– Ménière's disease	4 (8)	4 (8)
– Migraine	12 (24)	5 (10)
– PPPD	3 (6)	6 (12)
– Vestibular neuritis	4 (8)	3 (6)
– Mixed aetiology	–	7 (14)
– Other	16 (32)	12 (24)
Call completion rate	64% completed via video; 36% converted to phone calls	84% completed via phone; 16% converted to face-to-face
Discharged (*n* (%))	22 (44)	9 (18)
Requires face-to-face consult as next step (*n* (%))	10 (20)	22 (44)
Imaging requested (*n* (%))	5 (10)	5 (10)
VFTs requested (*n* (%))	7 (14)	13 (26)
PTA requested (*n* (%))	8 (16)	15 (30)

SD = standard deviation; BPPV = benign positional paroxysmal vertigo; PPPD = persistent postural perceptual dizziness; VFTs = vestibular function tests; PTA = pure tone audiometry

## Case report

A 61-year-old woman was referred with dizziness and imbalance. She had a background of left temporal bone meningioma with partial resection. Recent imaging scans of the brain and internal auditory meatus were available. Her audiology records showed that she had no useful hearing on the left side, with essentially normal hearing on the right. She had a left-sided bone-anchored hearing aid.

She was offered a video remote consultation for an assessment. At the beginning of the call, she confirmed she could hear and see the doctor adequately and her supporting family member was introduced.

She gave a history of dizziness presenting with a fall some years ago. Subsequent investigation had uncovered the meningioma. She now experienced daily symptoms of dizziness, of variable intensity, with difficulty making quick turns, standing with eyes closed, and unsteadiness. She reported days where she experienced several hours of vertigo requiring her to rest in bed, associated with photophobia and phonophobia. She had daily headaches and a history of typical migraine in earlier adulthood.

Examination via the video link showed a full range of neck and eye movements without symptom provocation. She demonstrated impaired co-ordination for the left upper limb when compared to the right, along with a mild kinetic tremor. Under guidance, she carried out a side-lying test on a sofa. She showed adequate acceleration and positioning of the head with respect to gravity. She had minor symptoms on the left-ear-down position, without frank vertigo. She reported symptoms of light-headedness on sitting up from either side. She was able to stand unsupported with eyes closed for 5 seconds. She was observed walking and noted to be unsteady, especially on quick turns.

Because of the orthostatic symptoms observed in the positioning assessment, postural blood pressure measurements were appropriate. She did not have home sphygmomanometry available, but was able to obtain the equipment from elsewhere, follow a lying–standing blood pressure assessment protocol, and send the results electronically along with her completed outcome measures.

This assessment identified: elements of a complex balance disorder with contributions from structural peripheral and central vestibular deficits, orthostatic intolerance, positional symptoms, and migrainous symptoms. She was given advice on positional and orthostatic symptom management, a starting point for a home exercise programme, and headache and migraine management. Vestibular function tests were planned as a face-to-face appointment, to take place in due course, along with a review of the above interventions.

## Discussion

The case illustrates important aspects of the remote neuro-otology consultation. The patient should be instructed to plan for an uninterrupted session, with no young children or pets present, as these not only distract but can also be a safety hazard during stance and gait assessments. Patients also need to consider how to set the camera in a location that will enable demonstration of gait and stance safely, with a suitable room, furniture placement, wall corner and chair positioning in front of the patient for safety. Some testing, especially positional testing, is easier if a family member can be present.

The clinician must ensure that there is a contingency plan in case urgent examination or care is required. As in the face-to-face clinic, patients with dizziness may well experience symptoms during a consultation and the clinician should be prepared to handle this situation, knowing the exact location of the patient and who else is on hand.

The history can be covered by telephone or video in exactly the same way that it is done in the clinic. In a video consultation, some elements of physical examination can also be conducted, as illustrated in this case.

Eye movements can be examined through the resolution limits of the technology by asking the patient to bring their eyes close to the camera, and establishing the range of gaze in all directions. Nystagmus may be visible in some cases. Facial nerve function and tongue movements, and upper limb co-ordination tests such as finger–nose co-ordination and rapid alternating movement can be conducted. Stance with eyes open and eyes closed can be assessed in a room corner, with a chair placed in front of the patient to ensure stability and safety. Gait and tandem gait may be observed if safe to do so.

Side-lying positioning is possible with the patient sitting on a sofa or a bed, and the examiner may demonstrate via the video link how to obtain the required head and body position. Patients must, as in normal clinical practice, be warned that symptoms may be induced, and so clinical judgement is required as to the advisability of remote positioning. It is sensible to ensure the patient has suitable support from a friend, family member or carer, before remote positioning is attempted. Some patients may be able to hold a smartphone camera in a position that is able to record eye movement during positioning, or the help of another adult in the home may be enlisted.

We have successfully treated many cases of both lateral and posterior canal benign positional paroxysmal vertigo (BPPV) remotely. The use of a smart phone or tablet to record eye movements is helpful in case lateral canal BPPV is induced during an Epley manoeuvre. Having a family member to prevent head raise during the Epley manoeuvre may reduce this risk.

As stance, gait and positioning testing are more complex, it can be helpful to ask the patient to make videos of the required assessments, according to standardised instructions, which can be shared later for review. Home sphygmomanometry is not uncommon and, where available, instructions for postural blood pressure measurement may be given. The accuracy of any home measurements may need to be verified.

Some parts of the examination cannot be done remotely. Fundoscopy, head impulse testing, accurate assessment of smooth pursuit, saccades, and complete neurological examination of the upper and lower limbs are not possible.

- Coronavirus disease 2019 and other factors have driven interest in conducting remote consultations, but there has been little research on this topic in neuro-otology
- With suitable preparation, patients can have remote neuro-otological assessments that include elements of physical examination
- Neuro-otology units should look to install facilities for video consultations in preference to telephone consultations where possible

In many cases, as in our case illustration, information obtained remotely can be sufficient to make a working diagnosis, and management advice given. For example: online exercise programmes can be initiated, pending arrangements for a customised therapy programme; self-management of benign paroxysmal positional vertigo may be considered; and migraine management advice may also be offered. In the video consultation, there is the option of using the ‘chat’ function to communicate the names of medications, medical terms or diagnoses, or helpful online resource locations.

For many patients, the next steps will be clear, such as the requirement for investigation (e.g. magnetic resonance imaging, vestibular function tests, pure tone audiometry), or the need for a fuller physical examination (Table 1). This can then be arranged, and the patient can be reviewed in the clinic on only one occasion subsequently. In some cases, the clinician may have sufficient information to be able to safely discharge the patient or direct them to a more appropriate service, avoiding completely an unnecessary trip to hospital.

Table 1 provides quantitative data on experience with 100 patients. The telephone consultations and video consultations were conducted in different centres (London and Leicester), accounting for differences in the clinical populations. The video consultations were sometimes impaired by poor connectivity, and the telephone consultations were sometimes affected by language or communication barriers, or lack of patient preparedness for the consultation. The differing needs for further face-to-face review also reflect: the additional information that can be obtained from a video as opposed to the telephone; the different diagnostic mix between the two patient populations (e.g. migraine is easier to discharge than a multi-system balance disorder); the patient populations; and local protocols. However, the data give broad support for units running remote consultations to develop video rather than telephone capability in neuro-otology clinics where possible.

## Conclusion

There is a need for research studies to formally examine the diagnostic accuracy, safety and patient acceptability of remote consultations for neuro-otology, especially in light of the present Covid-19 pandemic. Such consultations have the potential to offer improved access, increased convenience and safety for patients, and to increase the efficient provision of neuro-otological care, both now and in the future.
